# Axon-glia interaction and membrane traffic in myelin formation

**DOI:** 10.3389/fncel.2013.00284

**Published:** 2014-01-06

**Authors:** Robin White, Eva-Maria Krämer-Albers

**Affiliations:** ^1^Institute of Physiology and Pathophysiology, University Medical Center of the Johannes Gutenberg UniversityMainz, Germany; ^2^Department of Molecular Cell Biology, Johannes Gutenberg UniversityMainz, Germany

**Keywords:** myelination, cell communication, Fyn kinase, membrane traffic, endocytosis, SNAREs, myelin disease, local protein synthesis

## Abstract

In vertebrate nervous systems myelination of neuronal axons has evolved to increase conduction velocity of electrical impulses with minimal space and energy requirements. Myelin is formed by specialized glial cells which ensheath axons with a lipid-rich insulating membrane. Myelination is a multi-step process initiated by axon-glia recognition triggering glial polarization followed by targeted myelin membrane expansion and compaction. Thereby, a myelin sheath of complex subdomain structure is established. Continuous communication between neurons and glial cells is essential for myelin maintenance and axonal integrity. A diverse group of diseases, from multiple sclerosis to schizophrenia, have been linked to malfunction of myelinating cells reflecting the physiological importance of the axon-glial unit. This review describes the mechanisms of axonal signal integration by oligodendrocytes emphasizing the central role of the Src-family kinase Fyn during central nervous system (CNS) myelination. Furthermore, we discuss myelin membrane trafficking with particular focus on endocytic recycling and the control of proteolipid protein (PLP) transport by soluble N-ethylmaleimide-sensitive factor attachment protein receptor (SNARE) proteins. Finally, PLP mistrafficking is considered in the context of myelin diseases.

## Introduction

In complex nervous systems, electrically excitable neuronal cells coexist with comparatively silent glial cells. The best understood principal function of glial cells is the formation of myelin by which conduction velocity of electrical impulses is increased in axons with relatively small diameters. Although myelin seems to have arisen independently several times in evolution and is found in vertebrate and invertebrate species, it shares remarkable morphological similarities (Hartline and Colman, 2007). Regardless of their source, myelinated axons are characterized by long segments of multilamellar membrane wrappings alternating with “nodal” gaps (nodes of Ranvier), where the molecular machinery for action potential generation resides and allows the saltatory mode of conduction. Reflecting the more ancestral function of ensheathing cells, vertebrate myelinating cells maintain the integrity of axons by providing glial support (Nave, 2010), which was suggested to include the transfer of glycolytic energy metabolites (Fünfschilling et al., 2012; Lee et al., 2012) and cargo-containing vesicles termed exosomes (Frühbeis et al., 2013; Lewis, 2013).

Myelin is formed as an extension of the plasma membrane of oligodendrocytes in the central nervous system (CNS) and Schwann cells in the peripheral nervous system (PNS; Sherman and Brophy, 2005). Their embryonic origin from neuroepithelial sources and subsequential specification mediated by the action of transcription factors as well as the parameters influencing their migration and differentiation have been well defined over the past few years (Colognato and Ffrench-Constant, 2004; Rowitch, 2004; Jessen and Mirsky, 2005, 2008; Kessaris et al., 2006; Wegner, 2008; Emery et al., 2009). CNS invasion by oligodendrocyte precursor cells (OPCs) and the dynamics of process elaboration as well as myelination has been visualized in the developing zebrafish by *in vivo* time-lapse imaging (Kirby et al., 2006; Czopka et al., 2013). Diffusible and contact-dependent neuronal signals are essential for the correct timing of glial differentiation. They regulate glial proliferation and survival in order to match the numbers of glial cells to axons and specify which axons are myelinated (Barres and Raff, 1999; Simons and Trajkovic, 2006). While Schwann cell myelination occurs at a 1:1 ratio, oligodendrocytes myelinate up to 50 axonal segments simultaneously demanding a high level of cell organization. This requires CNS glial cells to be more versatile and their acquisition of additional mechanisms to adapt their behavior to environmental cues. The myelination programme is initiated in response to axon-glia recognition largely mediated by membrane bound cell adhesion molecules triggering reorganization of the glial cytoskeleton and cell polarization (Simons and Trotter, 2007; Bauer et al., 2009). A balance of inhibiting and promoting molecules finally regulates myelin formation (Emery, 2010).

In humans and rodents, myelination in the CNS largely occurs during early postnatal development and can still proceed in the adult (Miller et al., 2012; Young et al., 2013). Spiral ensheathment of axons with multiple layers of glial membrane is followed by membrane compaction and formation of distinct adaxonal, abaxonal, and paranodal subdomains (Arroyo and Scherer, 2000). The myelin membrane has a highly unique protein and lipid composition and its subdomains exhibit a characteristic molecular architecture. In particular, the paranodal junctions formed between myelin and the axon membrane represent highly structured multimeric protein complexes (Poliak and Peles, 2003; Schafer and Rasband, 2006). To establish such an elaborate membrane system, myelinating cells require an appropriate membrane sorting and trafficking machinery allowing temporal and spatial control by environmental cues (Baron and Hoekstra, 2010; Simons et al., 2012). Only recent work is beginning to reveal insight into the mechanistic link between axon-glia recognition, signaling and myelin membrane assembly (Aggarwal et al., 2011a). Such advances are desperately needed to understand the pathomechanisms of dysmyelination and to deal with the problem of inefficient remyelination responsible for the irreversible clinical course of myelin diseases. Here, we focus on major achievements in deciphering axonal signal integration and myelin membrane traffic in oligodendrocytes. Moreover, implications for the pathology of dysmyelinating diseases characterized by mistrafficking of the major myelin protein proteolipid protein (PLP) are discussed.

## Preparing glial cells for myelination

During CNS development, OPCs acquiring stable axonal contact differentiate into myelinating oligodendrocytes. A key event controlling the entrance of OPCs into the myelinating fate is the specific axon-glia recognition, apparently mediated by a number of surface-localized cell adhesion molecules and signaling receptors with seemingly redundant functions, possibly contributing to the remarkable robustness of myelination towards genetic ablation. Myelination implies a high degree of specificity and is regulated by axon size as well as repulsive and permissive signals (Simons and Lyons, 2013). Moreover, myelin formation depends on neuronal electrical activity (Demerens et al., 1996) at least partly mediated by the release of neurotransmitters such as ATP and glutamate along axons, promoting oligodendroglial differentiation and myelination (Stevens et al., 2002; Ishibashi et al., 2006; Wake et al., 2011). The manifold signals received from neurons have to be integrated by the glial cells to control timed differentiation, which involves cytoskeletal reorganization and cell polarization towards the axon-glia contact site, essential to prepare the cells for myelin formation.

**Figure 1:**
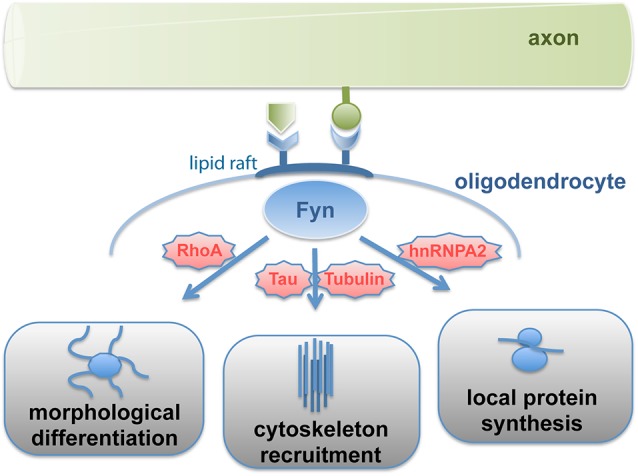
**Signal-transmission by Fyn kinase**. Fyn kinase associated with lipid rafts is activated by axon-derived soluble or membrane-bound signals, which relay into three major pathways regulating differentiation, cytoskeleton stability and cell polarization as well as local protein synthesis. Fyn thus integrates axonal signals to spatiotemporally regulate myelin formation.

Work over the last decade revealed that the non-receptor Src-family tyrosine kinase Fyn functions as an integrator of neuronal signals regulating the morphological differentiation of oligodendrocytes. Fyn kinase activity peaks during the initiation of myelination (Umemori et al., 1994; Krämer et al., 1999) and Fyn-deficient mice are characterized by abnormal oligodendrocyte development and hypomyelination (Sperber et al., 2001; Goto et al., 2008). On the cellular level, Fyn-inactivation interferes with oligodendroglial maturation and in particular process outgrowth (Osterhout et al., 1999; Sperber and Mcmorris, 2001). Various upstream activators such as *α*6*β*1-integrin, deleted in colorectal carcinoma (Dcc), Lingo-1 and the GPI-anchored protein F3/contactin have been identified, which sense axonal membrane-associated or soluble ligands and modulate Fyn-activity (reviewed in Krämer-Albers and White, 2011). Fyn signaling feeds into three major downstream pathways (Figure 1), which regulate Rho-family GTPase signaling and the morphological differentiation of oligodendrocytes (Liang et al., 2004), the recruitment of microtubule cytoskeleton components (Klein et al., 2002), and the local translation of the myelin protein MBP (White et al., 2008).

Fyn kinase activity in response to F3/contactin stimulation is compartmentalized into lipid raft membrane domains, which are formed during oligodendrocyte maturation (Krämer et al., 1999). These dynamic sterol- and sphingolipid-enriched liquid ordered membrane microdomains recruit specific proteins (such as F3/contactin, *α*6*β*1- integrin and Fyn) and promote their interaction in signaling pathways. Fyn-activation causes the protein to adopt an open conformation rendering its SH2- and SH3-domains accessible to specific downstream targets. These domains mediate for instance the binding of Tubulin and the microtubule associated protein Tau, which regulates the assembly and stabilization of microtubules (Gorath et al., 2001; Richter-Landsberg, 2008). Interference with Fyn-Tau interaction impairs oligodendroglial process outgrowth and myelination despite normal Fyn activity levels (Klein et al., 2002; Belkadi and Lopresti, 2008). Thus, oligodendroglial lipid rafts appear to function as signaling platforms linking cell adhesion to cell polarization by recruitment of microtubule cytoskeleton components to sites of contact-mediated Fyn activation.

Fyn activation in response to binding of the axonal cell adhesion molecule L1 facilitates the site-specific translation of myelin basic protein (MBP; White et al., 2008). MBP is a peripheral membrane protein that is required for myelin formation (Readhead et al., 1990). It is supposed to promote compaction of the myelin membrane by electrostatic interaction of its basic amino acids with negatively charged membrane interfaces. MBP translation occurs localized at active sites of myelin synthesis (Colman et al., 1982; Carson et al., 2008). The mRNA of MBP binds to the transacting factor heterogeneous nuclear ribonucleoprotein A2 (hnRNP A2) and is packed into RNA-granules together with components of the translation machinery in a translationally repressed state. Translational repression of MBP mRNA is regulated by hnRNP E1 and the small non-coding RNA 715 (sncRNA715; Kosturko et al., 2006; Bauer et al., 2012). The granules are transported to distal sites of oligodendroglial processes where MBP translation is initiated in response to L1/contactin-mediated Fyn activation, which triggers phosphorylation of hnRNP A2 and other granule components such as hnRNP F resulting in granule dissociation (White et al., 2008, 2012). The laminin-2 receptor *β*1-integrin (Colognato et al., 2002) is associated with Fyn by interacting with the L1-receptor F3/contactin in the oligodendroglial membrane in *cis* and appears to cooperate in this process by recruiting the RNA-binding protein hnRNP K (Colognato et al., 2004; Laursen et al., 2009, 2011). In addition, Fyn phosphorylates the MBP mRNA binding protein QKI, which regulates MBP mRNA stability (Lu et al., 2005). Intriguingly, Fyn-dependent MBP translation appears to be coupled to axonal action potential firing and glutamate signaling (Wake et al., 2011).

Thus, oligodendroglial Fyn integrates axonal and extracellular matrix-derived signals such as laminin-2 to control timed differentiation and localized synthesis of myelin, thereby adapting myelin formation to neuronal properties. From that perspective, it may appear surprising that Fyn-deficient mice are still capable of forming myelin, though at reduced levels. Recent work revealed the PI3-Kinase/Akt/mTOR and ERK1/2-MAPK pathways as well as CAMKII*β* signaling as important regulators of myelin growth (Flores et al., 2008; Goebbels et al., 2010; Ishii et al., 2013; Waggener et al., 2013). Crosstalk between these pathways may compensate for the loss of individual signaling components and prevent stronger defects in myelin synthesis.

## Myelin biogenesis: membrane trafficking and the role of endocytic recycling

Following axon recognition and positioning of myelination-competent oligodendrocytes, the onset of myelin biogenesis requires the setup of an intricate membrane sorting and trafficking machinery. The highly specialized myelin membrane has a unique composition of proteins and lipids, which need to be sorted and directed to the sites of myelin membrane growth. Sorting of myelin components has been suggested to occur in the Golgi-apparatus (Simons et al., 2000), by endocytic recycling (Winterstein et al., 2008), and at the level of the plasma membrane (Aggarwal et al., 2011b). The assembly of myelin seems to underlie at least partially the biophysical principles of self-organizing systems (Aggarwal et al., 2011a). Compared to normal plasma membranes, myelin is very lipid-rich (70% of myelin dry weight). The myelin-specific glycosphingolipids galactocerebroside and sulfatide exhibit biophysical characteristics favoring self-assembly with cholesterol and certain myelin proteins into raft-like membrane microdomains. Lipid rafts enriched in these myelin lipids are formed in the Golgi-apparatus of maturing oligodendrocytes (Krämer et al., 1997; Simons et al., 2000; Gielen et al., 2006). One of the main raft components is the major myelin protein PLP, a highly hydrophobic tetraspan protein that is strongly palmitoylated and binds cholesterol. Thus, in addition to their role in Fyn-signaling upon axon-glia interaction (see above), raft-like microdomains mediate sorting of myelin components. Further evidence suggests that raft-like protein-lipid interactions have a distinct function in the formation and maintenance of the paranodal junctions (Schafer et al., 2004). Taken together, myelin proteins and lipids laterally associate into preformed myelin elements during transport through the secretory pathway reflecting a primary step in myelin assembly. Upon arrival at the plasma membrane, these preformed myelin elements need to be coalesced to build up the myelin sheath.

Recent work in cultured oligodendrocytes indicates that locally translated MBP organizes the formation of membrane sheets characteristic of compact myelin. The basic MBP carries positive charges that interact with the negatively charged headgroups of inner leaflet phospholipids such as phosphatidylinositol-(4,5)-bisphosphate mediating a higher lipid order and condensation of the two opposing cytoplasmic leaflets (Fitzner et al., 2006; Musse et al., 2008; Nawaz et al., 2009). There is evidence that MBP glues together the adjacent membranes by self-association into higher order aggregates creating a meshwork that furthermore acts as a physical filter restricting the diffusion of proteins with large cytoplasmic domains into the compact myelin domain (Aggarwal et al., 2011b, 2013). Thus, MBP appears to reinforce the coalescence of myelin membrane domains and the segregation of compacted versus non-compacted membranes. These functional properties of MBP may explain the necessity of local MBP translation to avoid the premature clustering and compaction of membranes at inappropriate intracellular sites. Notably, MBP is subject to various posttranslational modifications modulating its charge level, which may regulate its ability to promote sheet segregation and membrane compaction (Harauz and Musse, 2007).

**Figure 2:**
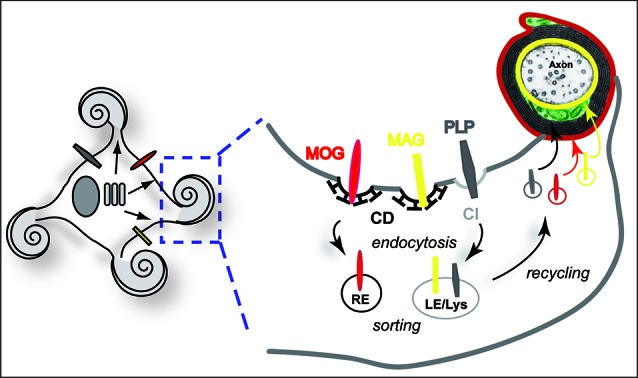
**Endocytic recycling of myelin proteins and oligodendroglial membrane remodeling**. Schematic illustration of endocytic sorting and recycling of myelin proteins into distinct domains of the myelin membrane. MAG (yellow) and MOG (red) are internalized by CD endocytosis, while PLP (dark gray) utilizes a CI, cholesterol-dependent pathway. PLP and MAG are sorted to LE/Lys, while MOG resides in RE. The model suggests recycling to their final location in distinct myelin domains. Local storage and recycling of myelin components may favor simultaneous myelin formation at multiple internodal segments.

In addition to segregation of myelin components, myelin biogenesis requires the operation of directed vesicular trafficking pathways. Oligodendrocytes possess a complex architecture and myelin is organized in subdomains. Due to spatial restrictions, vesicular transport is largely confined to the cell processes and the non-compact cytoplasmic channels of the myelin sheath. The observation that PLP is enriched in late endosomes/lysosomes (LE/Lys) has put forward the idea that endocytic recycling may contribute to myelin biogenesis (Krämer et al., 2001; Trajkovic et al., 2006). In the brain, late endosomal localization of PLP is particularly evident during active myelinogenesis (Bakhti et al., 2011). Intriguingly, endocytosis of PLP and its subsequent recycling to the plasma membrane is regulated by a soluble neuronal signal controlling the small GTPase RhoA (Trajkovic et al., 2006; Kippert et al., 2007). Downregulation of RhoA activity facilitates the redistribution of PLP from late endocytic pools to the plasma membrane. The mobilization of PLP-containing LE/Lys was also dependent on c-Src, which localized to LE/Lys in its active form. In addition to PLP, myelin-associated glycoprotein (MAG) and myelin-oligodendrocyte glycoprotein (MOG) also recycle through endocytic compartments, although they utilize distinct pathways (Winterstein et al., 2008). While PLP is endocytosed by clathrin-independent (CI) endocytosis, MAG and MOG utilize the clathrin-dependent (CD) pathway but are differently targeted to LE/Lys and recycling endosomes (RE), respectively (Figure 2). Differential endocytic sorting of MAG, MOG, and PLP may be linked to their distinct localization to the adaxonal loop, abaxonal loop, and compact domain of myelin. Consistent with this idea, recycling through endosomes in cultured oligodendrocytes promotes the transition of MAG, MOG, and PLP to membrane domains that already define the characteristics of their final subdomain in myelin.

Endocytic recycling thus promotes oligodendroglial membrane remodeling, which could drive the morphogenesis of the distinct domains of compact and non-compact myelin. Site-specific membrane removal and targeted re-addition is commonly utilized by cells to establish polarized membrane domains and to mediate rapid directed membrane growth (Lecuit and Pilot, 2003; Shivas et al., 2010). The establishment of distal endosomal membrane reservoirs in myelinating cells may be critical for myelin assembly in the cell periphery,** which in oligodendrocytes occurs simultaneously at multiple sites. The versatile endosomal system is connected to the exocytic as well as to the lysosomal degradative machinery and thus has the capability to control homeostatic myelin production and turnover. It is likely that these processes are regulated by axonal signals adapting myelin biogenesis to parameters such as axonal diameter or internodal length. However, the neuronal signal controlling Rho-activity and recycling through LE/Lys remains undefined. It may be interesting to further explore whether axon-glia interaction involving integrin signaling to Fyn and Rho regulates endocytic recycling.

Taken together, the findings endorse the following model of myelin membrane traffic: newly synthesized myelin components reach the plasma membrane after initial lipid-raft-mediated self-assembly in the Golgi apparatus largely by non-targeted vesicular transport. The compact myelin domain forms at the level of the plasma membrane by lateral coalescence of myelin components guided by MBP, which acts as a diffusion barrier. Endosomal pools of myelin components in the periphery serve as membrane reservoirs and sorting stations from which myelin components can rapidly be mobilized to local sites of myelin synthesis. Furthermore, endocytic recycling assists membrane remodeling regulating the precise spatiotemporal targeting of myelin proteins essential for polarized myelin membrane growth and subdomain formation.

## Control of endosomal traffic and transport of proteolipid protein (PLP) by soluble N-ethylmaleimide-sensitive factor attachment protein receptors (SNAREs)

**Figure 3:**
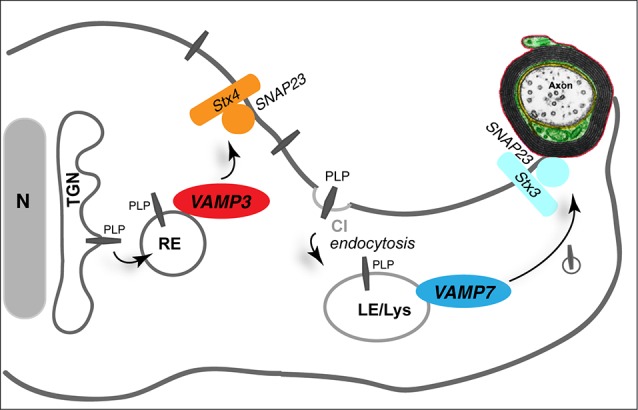
**Role of SNAREs in myelin membrane traffic**. The R-SNAREs VAMP3 and VAMP7 control surface transport of PLP. VAMP3 (red) mediates fusion of recycling endosome (RE)-derived vesicles with the plasma membrane, where Syntaxin 4 (Stx4) and SNAP23 act as cognate target-SNARES (orange). VAMP7 (dark blue) interacts with target SNAREs Syntaxin 3 (Stx3) and SNAP23 (light blue) to control transport from LE/Lys to the myelin membrane. TGN, trans-Golgi network.

Myelin membrane traffic requires targeted membrane fusion. How do myelinating cells achieve the site-specific fusion of vesicles transporting myelin components required for the expansion of the myelin membrane? The core of the eukaryotic membrane fusion machinery is formed by a complex of SNARE-proteins, which constitute a family of conserved proteins in eukaryotes and operate by specific pairing of vesicle (R)- and target (Q)-SNAREs (Jahn and Scheller, 2006; Sudhof and Rothman, 2009). Individual combinations of R- and Q-SNAREs mediate specific fusion reactions at distinct subcellular locations (McNew et al., 2000; Scales et al., 2000). A comprehensive analysis of the SNARE expression profile in maturing oligodendrocytes revealed a map of R- and Q-SNAREs participating in putative fusion complexes (Feldmann et al., 2009). Consistent with the engagement of PLP in endocytic recycling processes, the R-SNAREs VAMP3/cellubrevin and VAMP7/TI-VAMP co-localize with PLP in RE and LE/Lys, respectively. Functional ablation of VAMP3 and VAMP7 in cultured oligodendrocytes interferes with cell surface transport of PLP and impedes its association with myelin-like membranes in oligodendrocyte-neuron co-cultures, in which oligodendrocytes have started ensheathing axons (Feldmann et al., 2011). VAMP3 and VAMP7 act synergistically in PLP transport and appear to control fusion within independent transport pathways. Furthermore, they interact with distinct Q-SNARE acceptor complexes in the target membrane: VAMP3 pairs with syntaxin-4 and SNAP23, while VAMP7 binds to syntaxin-3/SNAP23, equivalent to polarized epithelial cells, where the same complexes control fusion of apical and basolateral cargos, respectively (Carmosino et al., 2010). Thus, VAMP3 appears to mediate plasma membrane fusion of vesicles originating from the recycling endosome (probably in the course of a biosynthetic route from the Golgi to the plasma membrane), while VAMP7 controls fusion of LE/Lys-derived vesicles (Figure 3).

*In vivo*, VAMP3 and VAMP7 are not essential for myelin formation per se. VAMP3-deficient mice as well as AP3-*δ* mutant *mocha* mice, which lack VAMP7-decorated LE/Lys, exhibit normal levels of myelination (Feldmann et al., 2011). However, analysis of myelin composition revealed that *mocha* mice exhibit a mild dysmyelination characterized by reduced levels of selected myelin proteins such as PLP and 2′3′-cyclic nucleotide 3′-phosphodiesterase (CNP) in myelin, suggesting that VAMP7 is involved in directing these proteins to myelin. Thus, VAMP7-dependent fusion of lysosome-related organelles appears to mediate the delivery of specific components to the myelin membrane without affecting the wrapping process of myelination. Most likely, functional redundancy between VAMP3 and VAMP7 prevents stronger dysmyelination phenotypes in these mice. It may be anticipated that combined absence of VAMP3 and VAMP7 will provoke larger defects of myelin formation.

## Role of membrane traffic in myelin disease

Myelin diseases are characterized by a developmental dysmyelination (often presenting as hypomyelination) or a secondary demyelination. They can be inherited (leukodystrophies) or develop in adulthood, such as multiple sclerosis, which is an immune-mediated degeneration of the myelin sheath (Compston and Coles, 2008; Perlman and Mar, 2012). Since myelin membrane transport involves the secretory pathway, myelinating cells of the CNS and PNS are prone to errors in membrane traffic and in particular Endoplasmic Reticulum (ER) stress initiated by misfolded proteins (Lin and Popko, 2009).

Mutations in the X-linked *PLP1*-gene (encoding PLP and its smaller isoform DM20) lead to dysmyelinating disease with a broad clinical spectrum, ranging from severe Pelizaeus–Merzbacher disease (PMD) to milder spastic paraplegia type 2 (SPG-2). Mutations include duplications, missense and nonsense mutations, as well as deletions, which have been extensively studied in heterologous expression systems and in animal models (Garbern, 2007; Woodward, 2008; Gruenenfelder et al., 2011). The molecular pathology of PLP-related disorders has been generally attributed to ER-retention of misfolded PLP and activation of the unfolded protein response (UPR), however, the genotype-phenotype relationship is less clear. While oligodendrocyte apoptosis is a hallmark of severe disease, the cause for dysmyelination in the milder cases is not well understood. Analysis of PLP/DM20 trafficking in oligodendroglial cells revealed that missense mutations associated with mild and severe disease exhibit distinct trafficking defects (Krämer-Albers et al., 2006). Mutant *jimpy-msd* PLP (A242V) causing severe PMD is arrested in the ER, while mutant *rumpshaker* PLP (I186T) associated with milder SPG2 reaches the LE/Lys. The interaction of both mutant proteins with cholesterol and lipid rafts is impaired. Furthermore, their turnover is increased, indicating that intracellular protein accumulation is not part of the pathology. Dysmyelination is not caused by the loss of PLP function, as *PLP1* deletion does not affect myelination. These studies suggest that missense mutations associated with a severe phenotype are mainly characterized by a complete transport arrest of PLP/DM20 and induction of the UPR followed by oligodendrocyte cell death, while mutations associated with milder phenotypes allow escape of mutant PLP from the ER but lead to dysmyelination possibly due to its deficiency to bind cholesterol and sort into lipid rafts. Myelin is rich in cholesterol and the availability of cholesterol is known to be a rate-limiting factor of myelination (Saher et al., 2005). Another critical parameter of PLP surface transport is the correct intramolecular formation of disulfide bridges and the proper self-alignment of its transmembrane domains (Dhaunchak and Nave, 2007; Dhaunchak et al., 2011). ER-retention of a subgroup of mutations in the extracelluar loop region can be reverted by removal of specific cysteines.

In more than half of all PMD cases, the patients carry a duplication of the *PLP1* gene. Overexpression of wild-type PLP leads to the sequestration of cholesterol and other lipid raft components away from the plasma membrane into LE/Lys, which was suggested to perturb myelination (Simons et al., 2002). Trafficking of PLP through late endosomal compartments also involves its association with multivesicular bodies (MVBs) and the intraluminal vesicles inside (Krämer-Albers et al., 2007). These MVBs can adopt an exocytic fate and fuse with the plasma membrane resulting in vesicle release into the extracellular space. These endosome-derived secreted vesicles are termed exosomes (Raposo and Stoorvogel, 2013) and recent evidence indicates that exosomes are engaged in mutual neuron-glia communication (Frühbeis et al., 2012, 2013). It could be speculated that PLP overexpression and the sequestration of PLP and cholesterol into LE/Lys has a qualitative or quantitative impact on exosome secretion from oligodendrocytes, which could relate to the myelin degeneration and the axonopathy observed in *PLP1*-transgenic animals and PMD patients. Intriguingly, a high cholesterol diet applied to transgenic mice carrying extra copies of *PLP1* ameliorated intracellular accumulation of PLP and facilitated myelin formation (Saher et al., 2012), suggesting that the stoichiometric relationship between PLP and cholesterol is important for the mobilization of PLP from the late endocytic pools and proper myelin membrane trafficking.

## Conclusions

Myelinated axons constitute a functional entity that is built for life and relies on continuous mutual interaction between axons and glial cells. These interactions control oligodendrocyte survival, differentiation and homeostasis. The mechanisms of myelin biogenesis and maintenance are still not completely understood but important advances have been made in deciphering trafficking pathways and translational control of myelin proteins, as well as myelin assembly. The future challenge will be to decipher the interplay between these processes, to define the sites of myelin membrane elongation and, furthermore, the axonal signals influencing these pathways. Though the key role of Fyn as signal integrator has been recognized, further efforts are required to reveal its full potential in regulating myelin membrane biogenesis.

## Conflict of interest statement

The authors declare that the research was conducted in the absence of any commercial or financial relationships that could be construed as a potential conflict of interest.

## Abbreviations

CNP: 2′3′-cyclic nucleotide 3′-phosphodiesterase
CNS: central nervous system
ER: endoplasmic reticulum
hnRNP: heterogeneous nuclear ribonucleoprotein
LE/Lys: late endosomes/lysosomes
MAG: myelin associated glycoprotein
MBP: myelin basic protein
MOG: myelin oligodendrocyte glycoprotein
MVB: multivesicular body
NRG1: neuregulin-1 type III
PLP: proteolipid protein
PMD: Pelizaeus Merzbacher Disease
PNS: peripheral nervous system
SNARE: soluble N-ethylmaleimide-sensitive factor attachment protein receptor
SPG-2: Spastic Paraplegia-Type 2
TGN: trans golgi network
UPR: unfolded protein response
VAMP: vesicle associated membrane protein.
